# Sustained low functional impairment in axial spondyloarthritis (axSpA): which are the primary outcomes that should be targeted to achieve this?

**DOI:** 10.1186/s13075-023-03055-1

**Published:** 2023-04-28

**Authors:** Walter P. Maksymowych, Robert D. Inman, Louis Bessette, Proton Rahman, Emmanouil Rampakakis, Odalis Asin-Milan, Meagan Rachich, Anne Marilise Marrache, Allen J. Lehman

**Affiliations:** 1 Department of Medicine, Division of Rheumatology, University of Alberta, 568 Heritage Medical Research Building, T6G 2S2 Edmonton, Canada; 2 CARE Arthritis, Edmonton, Canada; 3 Schroeder Arthritis Institute, University Health Network, University of Toronto, Toronto, ON Canada; 4 Department of Medicine, Université Laval, Quebec, QC Canada; 5 Faculty of Medicine, Memorial University, St John’s, NL Canada; 6 Scientific Affairs, JSS Medical Research, Montreal, QC Canada; 7 Medical Affairs, Janssen Inc., ON Toronto, Canada

**Keywords:** Spondyloarthritis, Treat-to-target, Infliximab, Golimumab, TNFi, Function, BASDAI, BASFI, ASDAS

## Abstract

**Objectives:**

To (i) determine whether sustained disease activity states, as measured by Bath Ankylosing Spondylitis Disease Activity Index (BASDAI) and Ankylosing Spondylitis Disease Activity Score (ASDAS), impact function, and (ii) evaluate characteristics predicting sustained low functional impairment in a prospective axial spondyloarthritis (axSpA) cohort.

**Methods:**

Biologic Treatment Registry Across Canada (BioTRAC) was a multi-center, prospective registry that collected real-world data on axSpA patients receiving infliximab or golimumab between 2006 and 2017. Generalized estimating equations (GEE) were used to test baseline characteristics, treatment, and duration (at 6 and 12 months vs. only at 6 or 12 months vs. neither) of low BASDAI (< 3), ASDAS-inactive disease (ID)(< 1.3), and ASDAS-low disease activity (LDA) in predicting sustained low Bath Ankylosing Spondylitis Functional Index (BASFI)(< 3) between 12 and 18 months. The adjusted impact of achieving low disease state at 6 and/or 12 months on BASFI at 18 months was analyzed by generalized linear models.

**Results:**

Eight hundred ten patients were enrolled. 33.7%, 13.4%, and 24.7% achieved sustained low BASDAI, ASDAS-ID, and ASDAS-LDA, respectively. In univariable GEE of baseline variables, age and baseline BASDAI, BASFI, and ASDAS significantly predicted sustained low BASFI. In multivariable GEE, sustained low BASDAI (*p* < 0.001), low BASDAI only at 6 or 12 months (*p* = 0.001), and baseline BASFI (*p* < 0.001) were the only predictors of sustained low BASFI. Sustained ASDAS-ID (*p* = 0.040) and ASDAS-LDA (*p* < 0.001) were also predictors when forced into the model. Similar results were obtained when evaluating the BASFI score at 18 months.

**Conclusion:**

Sustained BASDAI < 3 may be a valid and feasible target for a treat-to-target strategy in axSpA having function as treatment goal.

**Supplementary Information:**

The online version contains supplementary material available at 10.1186/s13075-023-03055-1.

## Background

Axial spondyloarthritis (axSpA), comprising ankylosing spondylitis (AS) and non-radiographic axSpA, is a chronic inflammatory rheumatic disease affecting the axial skeleton [1, 2]. Although the main symptom of axSpA is inflammatory back pain, patients may present with variable levels of sacroiliac joint and spinal structural damage, especially spinal ankylosis, and this may impair spinal mobility [2]. In addition, extra-spinal manifestations such as peripheral arthritis, enthesitis, and extra-musculoskeletal features such as uveitis, psoriasis, and inflammatory bowel disease (IBD) may occur [2, 3]. As a result, axSpA may lead to functional impairment which, in addition to pain and fatigue, can interfere with social participation, work, and schooling [4, 5]. In early disease, inflammation is a key reversible factor that determines functional impairment, while in later disease, irreversible structural changes become more important [6, 7].

Current international recommendations for the management of axSpA call for the use of non-steroidal anti-inflammatory drugs (NSAIDs) as a first-line treatment followed by anti-tumor necrosis factor (TNF) agents and, more recently, interleukin (IL)-17A inhibitors [3, 8, 9], with the ultimate goal of controlling symptoms, preserving function, and preventing structural damage as much as possible. A treat-to-target (T2T) strategy has been promoted for the management of active axSpA; however, no consensus exists as to the most appropriate treatment goal that should be targeted for implementation of a T2T strategy [8–11]. Structural damage assessed by radiography is not a feasible therapeutic goal in axSpA for the evaluation of T2T strategies as reliable detection of change in spinal damage requires at least 2 years of follow-up [12]. Functional impairment, as measured using the Bath AS Functional Index (BASFI), is a responsive outcome measure that is used in both clinical practice and randomized placebo-controlled trials. The BASFI is a 10-item patient self-reported questionnaire that is highly feasible for clinical practice and research [13]. The BASFI score is associated with the degree of inflammation and structural damage and may therefore be an appropriate therapeutic goal for a T2T strategy in axSpA [7]. In particular, a BASFI score of < 3 has been reported to reflect a patient acceptable symptom state [14].

There is also no consensus as to which outcome(s) should be monitored/targeted in a T2T strategy, though there is agreement that the key domain is disease activity [11, 14]. The Bath AS Disease Activity Index (BASDAI) is a 6-item patient self-reported questionnaire that is used extensively as a measure of disease activity in clinical practice and research [15]. The AS Disease Activity Score (ASDAS) is a disease activity outcome that comprises items 2, 3, and 6 of the BASDAI, measuring spinal and peripheral joint pain and degree of morning stiffness, plus patient global evaluation of disease activity on a 0–10 scale and the serum C-reactive protein (CRP) level [16]. Cut-offs defining different levels of disease activity according to the ASDAS have been validated [17] and it has been recommended as the outcome that should be monitored in a T2T strategy for axSpA. In particular, it has been recommended that attainment of an ASDAS of less than 2.1, termed low disease activity (ASDAS-LDA) [18], or even an ASDAS of less than 1.3, termed ASDAS inactive disease (ASDAS-ID) would be desirable [9]. But the Bath AS Disease Activity Index (BASDAI) is easier to implement in routine clinical practice because the CRP is often not available at the time of the patient visit in routine clinical practice. A BASDAI score of < 3 has been reported to reflect a patient acceptable symptom state [14].

There is also the question whether maintenance of a low disease activity state over a prolonged period in axSpA is desirable, as documented for rheumatoid arthritis, where sustained low disease activity over at least 6 months is associated with improved functional and structural damage outcomes [19].

The objectives of the current study were to determine whether and to what degree sustained (≥ 6 months) low disease activity states, as measured by BASDAI < 3 and ASDAS (< 1.3 and < 2.1), impact function and which of these states was most impactful. We also aimed to explore patient and disease characteristics as predictors of sustained low functional impairment, focusing on a comparison of sustained low ASDAS, both ASDAS-ID and ASDAS-LDA, versus low BASDAI.

## Patients and methods

### Study design

The Biologic Treatment Registry Across Canada (BioTRAC, NCT00741793) [20] was a multi-center, prospective registry that collected real-world patient-reported, clinical, and laboratory data on axSpA patients initiating treatment with infliximab or golimumab between 2006 and 2017 or 2010 and 2017, respectively. Given the observational nature of BioTRAC, patient management, including the initiation and discontinuation of infliximab or golimumab, was based solely on the judgment of the treating clinicians. However, follow-up at 6-month intervals was recommended. Treatment was continuous and treatment termination led to discontinuation from the study. Patients were required to provide written informed consent prior to enrollment in the study. Ethics approval was obtained by a central Research Ethics Board (IRB Services, Ontario, Canada) for private practices and respective Research Ethics Boards for institutional sites. BioTRAC was conducted in accordance with the Declaration of Helsinki.

### Study population

In order to be eligible for BioTRAC, patients needed to have a rheumatologist diagnosis of axSpA, be either bio-naïve (2006) or with ≤ 1 prior biologic agent exposure (2006–2017) and be eligible to receive either infliximab or golimumab as per the Canadian Product Monographs [21, 22]. No radiological data was required to be eligible for the study. To minimize selection bias, consecutive patients were screened and invited to participate in the study if the eligibility criteria were met.

### Study variables

The following patient-reported, clinical, and laboratory outcomes were collected, per routine care, in the BioTRAC registry at baseline and every six months thereafter: AS Disease Activity Score (ASDAS), Bath AS disease activity index (BASDAI), Bath AS functional index (BASFI), health assessment questionnaire (HAQ), patient global assessment (PtGA), physician global assessment (MDGA), back pain, enthesitis (yes vs. no; based on the Spondyloarthritis Research Consortium of Canada [SPARCC] Enthesitis Index) [23], dactylitis, and acute phase reactants (CRP, erythrocyte sedimentation rate [ESR]).

For the purposes of this analysis, the main endpoints of interest were (i) BASFI at 18 months of treatment and (ii) low BASFI (< 3) at 12 and 18 months. The main exposures of interest were achievement of (i) low BASDAI (< 3) at 6 *and* 12 months, at only 6 or 12 months, or at neither time; (ii) ASDAS-inactive disease (ID) (< 1.3) at 6 *and* 12 months, at only 6 or 12 months, or at neither time; and (iii) ASDAS low disease activity (LDA) (< 2.1) at 6 *and* 12 months, at only 6 or 12 months, or at neither time.

### Statistical methods

Baseline characteristics and disease parameters were summarized using descriptive statistics, namely the mean and standard deviation for continuous variables and counts and proportions for categorical variables. Univariable generalized estimating equations (GEE) were used to test the impact of baseline age, disease duration, gender, smoking, enrollment period, prior biologic experience, anti-TNF agent, non-biologic disease-modifying anti-rheumatic drug (nbDMARD) use, NSAID use, steroid use, presence of enthesitis and dactylitis, CRP, ASDAS, BASDAI, and BASFI and follow-up (low BASDAI at 6 and/or 12 months, ASDAS-ID at 6 and/or 12 months) parameters on achievement of sustained low BASFI at 12 and 18 months. Parameters showing a statistical trend (defined as *p* < 0.1) in the univariable analysis were entered in multivariable GEE models; stepwise backward variable selection was used to derive a parsimonious model. The impact of achieving sustained low BASDAI or ASDAS-ID at 6 and/or 12 months on the BASFI score at 18 months were analyzed using generalized linear models adjusted for age, gender, baseline disease duration, and baseline BASFI score. An additional model was built where the interaction of normal CRP (< 5 mg/L) with low BASDAI at 6 and/or 12 months was included as a predictor of BASFI at 18 months. For both the GEE and generalized linear models mentioned above, a secondary analysis was run in which ASDAS-ID was replaced with ASDAS-LDA.

No imputation of missing data was employed for the main analysis, and all analyses were ‘as observed’. A sensitivity analysis was also conducted using non-responder imputation for missing data through 18 months.

Statistical analyses were conducted with SPSS 24.0 (SPSS Inc., Chicago, IL).

## Results

A total of 810 patients were included, with a mean (standard deviation, SD) age of 45.9 (SD 12.7) years and disease duration of 7.4 (10.1) years (Table 1). An approximately equal proportion of patients were treated with golimumab and infliximab. The majority of patients were male (60.9%) and biologic naïve (86.8%). At baseline, 20.9% were on concomitant treatment with nbDMARDs, 65.7% received concomitant NSAIDs, and 11.7% oral steroids. Mean (SD) disease activity scores were indicative of very high disease activity, namely 3.7 (1.2) for ASDAS and 6.2 (2.1) for BASDAI while mean (SD) BASFI score was 5.6 (2.5).

**Table 1:** Baseline characteristics

Parameter	*N* = 810
Age, years, mean (SD)	45.9 (12.7)
Disease duration, years, mean (SD)	7.4 (10.1)
Male gender, *n* (%)	493 (60.9%)
Current smoker, *n*/*N* (%)^a^	139/542 (25.6%)
Enrollment period, *n* (%)	
• 2006–2008	192 (23.7%)
• 2009–2012	173 (21.4%)
• 2013–2015	310 (38.3%)
• 2016–2017	135 (16.7%)
Biologic naïve, *n* (%)	703 (86.8%)
nbDMARD use, *n* (%)	169 (20.9%)
• Methotrexate	120 (14.8%)
• Sulfasalazine	49 (6.0%)
• Hydroxychloroquine	13 (1.6%)
• Azathioprine	7 (0.9%)
• Leflunomide	7 (0.9%)
NSAID use, *n* (%)	532 (65.7%)
Steroid use, n (%)	95 (11.7%)
Enthesitis, *n*/*N* (%)^b^	156/721 (21.6%)
Dactylitis, *n*/*N* (%)^c^	39/484 (8.1%)
CRP, mg/L, mean (SD)	18.6 (62.3)
ASDAS, mean (SD)	3.7 (1.2)
BASDAI, mean (SD)	6.2 (2.1)
BASFI, mean (SD)	5.6 (2.4)
Anti-TNF, *n* (%)	
• Golimumab	421 (52.0%)
• Infliximab	389 (48.0%)

*ASDAS* Ankylosing Spondylitis Disease Activity Score; *BASDAI* Bath Ankylosing Spondylitis Disease Activity Index; *BASFI* Bath Ankylosing Spondylitis Functional Index; *CRP* C-Reactive Protein; *nbDMARD* non-biologic disease-modifying anti-rheumatic drugs; *NSAID* non-steroidal anti-inflammatory drugs; *SD* standard deviation; *TNF* tumor necrosis factor

^a^Information was missing for 268 patients

^b^Information was missing for 89 patients

^c^Information was missing for 326 patients

**Fig. 1:**
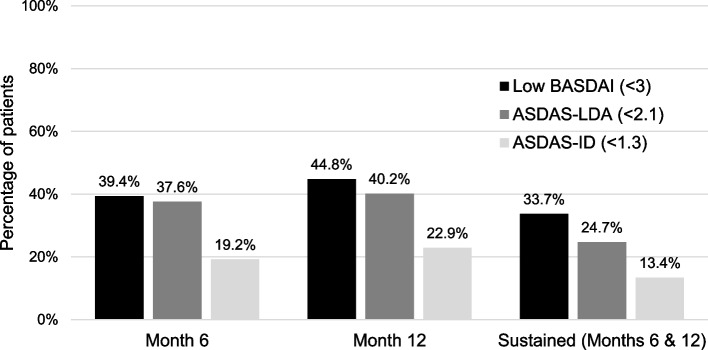
Measures of disease activity over time. Month 6 *n* = 626; month 12 *n* = 497; months 6 and 12 *n* = 457. BASDAI, Bath Ankylosing Spondylitis Disease Activity Index; ASDAS, Ankylosing Spondylitis Disease Activity Score; ID, inactive disease; LDA, low disease activity

Of the 810 patients enrolled, 626 attended the month 6 visit, 497 attended month 12, and 409 attended month 18. Achievement of low BASDAI, ASDAS-ID, and ASDAS-LDA at 6 months, 12 months, and sustained achievement at 6 and 12 months are shown in Fig. 1.

In univariable analysis of baseline demographics and disease parameters, higher age (OR [95%CI]: 0.96 [0.95, 0.98]), ASDAS score (0.6 [0.5, 0.8]), BASDAI score (0.7 [0.65, 0.8]), and BASFI score (0.66 [0.6, 0.7]) were identified as negative predictors of sustained low BASFI (< 3) between 12 and 18 months. In contrast, treatment with golimumab (1.6 [1.1, 2.2]) and achievement of low BASDAI, ASDAS-ID, and ASDAS-LDA at 6 and/or 12 months were positive predictors of sustained low BASFI (< 3) between 12 and 18 months (Table 2).

**Table 2:** Predictors of sustained low BASFI (< 3) between 12 and 18 months

Parameter	Univariable analysis		Multivariable analysis 1^a^ (*N* = 417)		Multivariable analysis 2 (*N* = 417)	
	OR (95% CI)	*P*-value	OR (95% CI)	*P*-value	OR (95% CI)	*P*-value
Age, years	0.96 (0.95, 0.98)	** < 0.001**	-	-	-	-
Disease duration, years	1.0 (0.98, 1.01)	0.335	-	-	-	-
Gender, male vs female	1.3 (1.0, 1.9)	0.087	-	-	-	-
Smoking	0.8 (0.5, 1.2)	0.230	-	-	-	-
*Enrollment period*		0.411	-	-	-	-
* 2006–2008 vs. 2016–2017*	0.7 (0.4, 1.2)					
* 2009–2012 vs. 2016–2017*	0.8 (0.4, 1.4)					
* 2013–2015 vs. 2016–2017*	1.0 (0.6, 1.6)					
Biologic naïve	1.0 (0.6, 1.6)	0.956	-	-	-	-
Baseline nbDMARD use	0.9 (0.6, 1.3)	0.530	-	-	-	-
Baseline NSAID use	1.1 (0.8, 1.6)	0.592	-	-	-	-
Baseline steroid use	1.2 (0.6, 2.3)	0.606	-	-	-	-
Anti-TNF, golimumab vs. infliximab	1.6 (1.1, 2.2)	**0.007**	-	-	-	-
Baseline enthesitis	0.9 (0.5, 1.4)	0.553	-	-	-	-
Baseline dactylitis	1.0 (0.4, 2.4)	0.967	-	-	-	-
Baseline CRP, mg/L	1.0 (0.99, 1.01)	0.257	-	-	-	-
Baseline ASDAS	0.6 (0.5, 0.8)	** < 0.001**	-	-	-	-
Baseline BASDAI	0.7 (0.65, 0.8)	** < 0.001**	-	-	-	-
BASFI, baseline	0.66 (0.6, 0.7)	** < 0.001**	0.7 (0.6, 0.8)	** < 0.001**	0.7 (0.6, 0.8)	** < 0.001**
Low BASDAI < 3						
* M6 and M12 vs. persistent BASDAI* ≥ *3*	37.3 (21.4, 64.9)	** < 0.001**	10.5 (4.0, 27.2)	** < 0.001**	7.4 (3.1, 17.7)	** < 0.001**
* M6 or M12 vs. persistent BASDAI* ≥ *3*	5.5 (3.3, 4.4)	** < 0.001**	5.2 (2.4, 11.1)	** < 0.001**	4.0 (1.8, 8.7)	**0.001**
ASDAS-ID (< 1.3)						
*M6 and M12 vs. persistent ASDAS* ≥ *1.3*	25.3 (9.2, 69.8)	** < 0.001**	3.7 (1.1, 13.1)	**0.040**	N/A	N/A
*M6 or M12 vs. persistent ASDAS* ≥ *1.3*	8.5 (3.8, 18.9)	** < 0.001**	2.2 (0.8, 6.0)	0.144	N/A	N/A
ASDAS-LDA (< 2.1)						
*M6 and M12 vs. persistent ASDAS* ≥ *2.1*	31.9 (14.6, 70.1)	** < 0.001**	N/A	N/A	7.2 (2.7, 19.3)	** < 0.001**
*M6 or M12 vs. persistent ASDAS* ≥ *2.1*	3.8 (2.0, 7.1)	** < 0.001**	N/A	N/A	2.1 (1.0, 4.2)	0.053

*BASFI* Bath Ankylosing Spondylitis Functional Index; *GEE* generalized estimating equation; *OR* odds ratio; *CI* confidence interval; *M* month; *BASDAI* Bath Ankylosing Spondylitis Disease Activity Index; *ASDAS* Ankylosing Spondylitis Disease Activity Score; *ID* inactive disease; *LDA* low disease activity

^a^Predictors retained following backward stepwise selection of parameters showing a *P* < 0.1 in univariable analysis. ASDAS-ID was forced into the model even though it was not retained. Bold *P*-values denote statistical significance

Multivariable analysis 1: primary analysis; multivariable analysis 2: secondary analysis including ASDAS-LDA instead of ASDAS-ID

In multivariable analysis, baseline BASFI and achievement of low BASDAI, whether sustained (i.e. at both 6 and 12 months) or at either 6 or 12 months (but no sustained), were the only significant predictors of low BASFI (< 3) between 12 and 18 months (Table 2); age, anti-TNF agent, baseline ASDAS score, baseline BASDAI score, and achievement of sustained ASDAS-ID over time, on the other hand, did not show a significant association with low BASFI. Both achievement of sustained low BASDAI (odds ratio, OR [95% confidence interval, CI]: 10.5 [4.0, 27.2]) and of low BASDAI at either 6 or 12 months (5.2 [2.4, 11.1]) were associated with significantly higher odds of sustained low BASFI between 12 and 18 months, though a greater effect was observed with the former. When forced in the multivariable model (Multivariable Analysis 1), sustained ASDAS-ID (3.7 [1.1, 13.1]), but not achievement of ASDAS-ID at either 6 or 12 months (2.2 [0.8, 6.0]), was associated with higher odds of sustained low BASFI between 12 and 18 months. In a secondary analysis using ASDAS-LDA instead of ASDAS-ID (Multivariable Analysis 2), achievement of sustained ASDAS-LDA, but not ASDAS-LDA at either 6 or 12 months, showed a significant association with sustained low BASFI between 12 and 18 months. A sensitivity analysis was also conducted using non-responder imputation for missing data which showed similar results to those described above (Supplementary Table 1).

Similarly, when evaluating BASFI at 18 months as a continuous outcome in multivariable analyses, age (B [95%CI]: 0.04 [0.02, 0.7]), baseline BASFI score (0.3 [0.2, 0.4]), and low BASDAI, both sustained (− 2.45 [− 3.35, − 1.55]) and at either 6 or 12 months (− 2.14 [− 2.88, − 1.41]), were strong predictors of BASFI score at 18 months, while sustained ASDAS-ID was a weaker predictor (Table 3; Model 1). In a secondary analysis using ASDAS-LDA instead of ASDAS-ID (Table 3; Model 2), sustained ASDAS-LDA, but not ASDAS-LDA at either 6 or 12 months, was a strong predictor of BASFI at 18 months (− 1.50 [− 2.36, − 0.64]), while age (0.04 [0.02, 0.7]), baseline BASFI score (0.29 [0.18, 0.4]), and low BASDAI, both sustained (− 2.22 [− 3.04, − 1.4]) and at either 6 or 12 months (− 1.91 [− 2.67, − 1.15]), continued to be strong predictors of BASFI score at 18 months.

**Table 3:** Predictors of BASFI score at 18 months in multivariable analyses

Model	Parameter	*B*^a^	95% CI	*P*-value
Model 1 (*N* = 180)	Baseline BASFI	0.30	0.19, 0.41	** < 0.001**
	Low BASDAI at M6 and M12 vs persistent BASDAI ≥ 3	− 2.45	− 3.35, − 1.55	** < 0.001**
	Low BASDAI at M6 or M12 vs persistent BASDAI ≥ 3	− 2.14	− 2.88, − 1.41	** < 0.001**
	ASDAS-ID at M6 and M12 vs persistent ASDAS ≥ 1.3	− 1.10	− 2.15, − 0.05	**0.041**
	ASDAS-ID at M6 or M12 vs persistent ASDAS ≥ 1.3	− 0.70	− 1.62, 0.22	0.136
	Age, years	0.04	0.02, 0.07	**0.001**
	Gender, male vs female	0.35	− 0.22, 0.92	0.228
	Baseline Disease Duration, years	− 0.02	− 0.05, 0.01	0.121
Model 2 (*N* = 180)	Baseline BASFI	0.29	0.18, 0.40	** < 0.001**
	Low BASDAI at M6 and M12 vs persistent BASDAI ≥ 3	− 2.22	− 3.04, − 1.40	** < 0.001**
	Low BASDAI at M6 or M12 vs persistent BASDAI ≥ 3	− 1.91	− 2.67, − 1.15	** < 0.001**
	ASDAS-LDA at M6 and M12 vs persistent ASDAS ≥ 2.1	− 1.50	− 2.36, − 0.64	**0.001**
	ASDAS-LDA at M6 or M12 vs persistent ASDAS ≥ 2.1	− 0.44	− 1.12, 0.25	0.213
	Age, years	0.04	0.02, 0.07	** < 0.001**
	Gender, male vs female	0.39	− 0.17, 0.96	0.172
	Baseline disease duration, years	− 0.02	− 0.05, 0.01	0.109

*BASFI* Bath Ankylosing Spondylitis Functional Index; *CI* confidence interval; *M* month; *BASDAI* Bath Ankylosing Spondylitis Disease Activity Index; *ASDAS* Ankylosing Spondylitis Disease Activity Score; *ID* inactive disease; *LDA* low disease activity

Bold *P*-values denote statistical significance

^a^Positive beta coefficient (B) indicates increased BASFI score at 18 months

Inclusion in the model of the interaction term low BASDAI by normal CRP (< 5 mg/L) at 6 and 12 months, showed a significant interaction (*P* = 0.046) by which sustained normal CRP was identified as an independent predictor of improved function among patients with sustained low BASDAI (− 2.14 [− 3.71, − 0.58]). No other significant interactions were observed.

## Discussion

A key contributor to quality of life for patients with axSpA is the preservation of physical function. In addition to enabling everyday tasks, physical function is vital for a patient’s ability to exercise, thus helping to prevent the development of comorbidities such as cardiovascular disease, which disproportionately affects patients with axSpA [11]. With this priority in mind, it may be suitable to structure treatment methodology around a goal of maintaining physical function while avoiding under- or over-treatment. A T2T approach targeting a measure of function may therefore be appropriate for patients with axSpA.

Although advocated by most international guidelines, to date, a T2T approach in axSpA lacks robust scientific evidence and is only indirectly supported by associations between levels of AS activity and future radiographic progression. It is for that reason that, in the latest update of the American College of Rheumatology/Spondylitis Association of America/Spondyloarthritis Research and Treatment Network recommendations for the treatment of axSpA [3], the panel judged that more convincing evidence of benefit should be present before endorsing this change in practice. In a longitudinal analysis of clinical measures of disease activity and radiographic progression that was adjusted for radiographic progression in the prior time interval of 2 years, all clinical measures were associated with progression, with little difference between the ASDAS or BASDAI analyzed as either continuous or categorical variables, except for the comparison of cases with very high ASDAS (> 3.5) versus ASDAS-ID [24]. It was stated that the longitudinal model with ASDAS as a continuous score had the lowest Quasilikelihood Information Criterion (QIC) of all models, even better than the model with BASDAI and CRP, indicating that the model with ASDAS as a disease activity measure best-captured variation in radiographic scores. However, the association of the ASDAS with radiographic progression was not significant in women or cases with symptom duration ≥ 18 years. Moreover, neither this nor other studies have compared the ASDAS and BASDAI (+ / − CRP) outcomes in a T2T strategy to assess the impact of sustained low disease activity on structural progression. Several observational studies evaluating anti-TNF agents for their potential to ameliorate radiographic progression have demonstrated that this can be achieved according to the level of control of inflammation although some studies have indicated that this may be accomplished by sustained normalization of acute inflammatory markers, ESR and CRP [25–28]. However, these studies were not designed with a T2T strategy aimed at attainment of a low disease activity state and did not directly compare the achievement of sustained ASDAS-ID with sustained low BASDAI.

The results of our study indicate that, in patients with axSpA treated with anti-TNFs, achievement of sustained low disease activity or remission within the first year was associated with significantly improved function at 18 months. Both sustained low BASDAI and ASDAS LDA were found to be strong predictors of improved function. Moreover, low BASDAI at either 6 or 12 months was a consistent predictor, but this was not the case for ASDAS-LDA. A limitation is that future time assessment at 18 months was not taken into account for disease activity (BASDAI, ASDAS) concurrently. Nonetheless, given that calculation of BASDAI does not include acute phase reactants and, thus, is more feasible in routine clinical care, this is an important finding that may have a significant impact on the application of T2T in daily practice. Achievement of sustained normal CRP among patients in sustained low BASDAI was found to be associated with further improvement in function, suggesting the importance of further lowering inflammation in these patients. This incremental benefit of CRP over and above low BASDAI may also be true for patients not achieving low BASDAI; however, this was not possible to evaluate due to the low number of patients that were not in sustained low BASDAI but were in sustained normal CRP.

Various cut-offs for defining a low disease activity state according to the BASDAI have been proposed such as < 2, < 3, and < 4. Similar cut-offs have been proposed for what constitutes an acceptable functional state. Some cut-offs have been validated against an external reference, this being most commonly the patient acceptable symptom state (PASS) question which asks patients whether they would globally consider their condition acceptable if they were to remain in their present state for the next month or the rest of their life [14, 29, 30]. These data showed that the value of the BASDAI cut-off that optimally reflects a yes response in receiver operating curve analysis varies according to age, gender, and symptom duration. Moreover, the optimal cut-offs become progressively lower with time after the start of treatment with an anti-TNF agent and it has been reported that these cut-offs plateau at 16 weeks with the cut-offs for both the BASDAI and BASFI being at 4–4.5 at the start of treatment and decreasing to 2.5–3.5 after 16 weeks of anti-TNF therapy. This data, in addition to BASDAI < 3 and BASFI < 3 having been reported to reflect a patient acceptable symptom state [14], was the rationale for our decision to choose a BASDAI of < 3 for defining low disease activity and BASFI < 3 for acceptable function. The use of a BASFI < 3 target has been reported previously in a 3-year long term extension of an anti-TNF RCT to demonstrate that attainment of sustained ASDAS-ID is associated with BASFI < 3 in all cases during years 2 and 3 of the follow up [31].

To our knowledge, this is one of the first studies directly comparing clinical measures of disease activity to determine which measures could be used for target monitoring in a T2T strategy in axSpA. In a previous pragmatic prospective, one-year trial, intensified treatment strategy aiming to achieve ASDAS < 2.1 was not associated with a significant benefit in terms of achieving a ≥ 30% improvement on the Assessment of SpondyloArthritis International Society (ASAS) Health Index (HI), the primary endpoint, compared to standard of care [32]; furthermore, although the T2T strategy was associated with significant benefits in terms of achieving ASAS40 and BASDAI50 responses, no significant differences in BASFI were observed between the study arms implementing T2T or usual care treatment strategies. An open-label phase III study evaluating the efficacy of a T2T strategy in bDMARD-naive patients with axSpA treated with secukinumab compared with standard-of-care treatment is ongoing [33]. The relationship between sustained ASDAS inactive disease over at least 2 consecutive time points 6 months apart, and structural lesion change has been evaluated using MRI erosion of the sacroiliac joints as a structural damage endpoint [34]. For the patients attaining sustained ASDAS inactive disease on etanercept, erosion decrease was evident in significantly more cases than erosion increase. However, the trend across ASDAS categories was not significant and a decrease in erosion was observed even in patients without a sustained ASDAS response.

Our study has certain limitations. The study was not designed to use a T2T protocol, even though T2T may have been used in routine care by some rheumatologists, or to be sufficiently powered to identify predictors with small to moderate effects which may have resulted in the non-detection of certain associations. Moreover, analysis of sustained endpoint achievement is limited in the context of a 4-visit, 18-month study. The analysis also does not include measures of Ankylosing Spondylitis Quality of Life (ASQoL) or ASAS HI. Furthermore, patient loss to follow-up may have resulted in selection bias, and radiographic progression was not evaluated. In addition, although the relative value of monitoring BASDAI and ASDAS was evaluated, a comprehensive assessment of other composite indices was not conducted; additional future studies will be required to evaluate the usefulness of other targets. Finally, although disease duration at baseline was not identified as a significant predictor of function, the findings may not be generalizable to patients with early disease.

## Conclusion

Aiming for sustained low BASDAI (< 3) may be a valid and feasible T2T treatment strategy for routine care in axSpA. Further validation of these real-world findings is required to aid in achieving consensus on which outcome should be monitored in a T2T strategy.

## Supplementary Information

**Additional file 1:** **Supplementary Table 1.** Predictors of Sustained Low BASFI (<3) Between 12 and 18 Months Using Non-Responder Imputation (Sensitivity Analysis). BASFI=Bath Ankylosing Spondylitis Functional Index; OR=Odds Ratio; CI=Confidence Interval; M=Month; BASDAI= Bath Ankylosing Spondylitis Disease Activity Index; ASDAS=Ankylosing Spondylitis Disease Activity Score; ID=Inactive Disease; LDA=Low Disease Activity. Bold P-values denote statistical significance. Multivariable Analysis 1: primary analysis; Multivariable Analysis 2: secondary analysis including ASDAS-LDA instead of ASDAS-ID.

## Data Availability

The data sharing policy of Janssen Pharmaceutical Companies of Johnson & Johnson is available at https://www.janssen.com/clinical-trials/transparency. As noted on this site, requests for access to the study data can be submitted through Yale Open Data Access [YODA] Project site at http://yoda.yale.edu.

## Abbreviations

AS: Ankylosing spondylitis
ASAS: Assessment of SpondyloArthritis International Society
ASDAS: AS Disease Activity Score
ASQoL: Ankylosing Spondylitis Quality of Life
axSpA: Axial spondyloarthritis
BASDAI: Bath AS Disease Activity Index
BASFI: Bath AS functional Index
BioTRAC: Biologic Treatment Registry Across Canada
CI: Confidence interval
CRP: C-reactive protein
ESR: Erythrocyte sedimentation rate
GEE: Generalized estimating equations
GLM: Generalized linear models
HAQ: Health assessment questionnaire
HI: Health Index
ID: Inactive disease
IL: Interleukin
LDA: Low disease activity
MDGA: Physician global assessment
nbDMARD: Non-biologic disease-modifying anti-rheumatic drug
NSAIDs: Anti-inflammatory drugs
OR: Odds ratio
PtGA: Patient global assessment
QIC: Quasilikelihood Information Criterion
SD: Standard deviation
SPARCC: Spondyloarthritis Research Consortium of Canada
T2T: Treat-to-target
TNF: Tumor necrosis factor
