# Supplementary material for: Sustained low functional impairment in axial spondyloarthritis (axSpA): which are the primary outcomes that should be targeted to achieve this?
Source: Arthritis Res Ther. 2023 Apr 28;25:70. doi: 10.1186/s13075-023-03055-1 (PMC10148455; doi:10.1186/s13075-023-03055-1)
Supplement: Supplementary file 1 — Additional file 1: Supplementary Table 1. Predictors of Sustained Low BASFI (<3) Between 12 and 18 Months Using Non-Responder Imputation (Sensitivity Analysis). BASFI=Bath Ankylosing Spondylitis Functional Index; OR=Odds Ratio; CI=Confidence Interval; M=Month; BASDAI= Bath Ankylosing Spondylitis Disease Activity Index; ASDAS=Ankylosing Spondylitis Disease Activity Score; ID=Inactive Disease; LDA=Low Disease Activity. Bold P-values denote statistical significance. Multivariable Analysis 1: primary analysis; Multivariable Analysis 2: secondary analysis including ASDAS-LDA instead of ASDAS-ID. [file 13075_2023_3055_MOESM1_ESM.docx]

**Supplementary Table 1. Predictors of Sustained Low BASFI (<3) Between 12 and 18 Months Using Non-Responder Imputation (Sensitivity Analysis)**

| **Parameter** | **Multivariable**  **Analysis 1**  **(N=810)** | | **Multivariable**  **Analysis 2**  **(N=810)** | | |
| --- | --- | --- | --- | --- | --- |
|  | OR (95% CI) | P-Value | OR (95% CI) | P-Value |  |
| Low BASDAI<3  *M6 and M12 vs.*  *persistent BASDAI ≥3*  *M6 or M12 vs.*  *persistent BASDAI ≥3* | 24.1 (16.0, 36.4)  5.5 (3.6, 8.5) | **<0.001**  **<0.001** | 21.6 (14.6, 31.9)  5.0 (3.2, 7.9) | **<0.001**  **<0.001** |  |
| ASDAS-ID (<1.3)  *M6 and M12 vs.*  *persistent ASDAS ≥1.3*  *M6 or M12 vs.*  *persistent ASDAS ≥1.3* | 1.9 (0.8, 4.6)  1.4 (0.7, 3.1) | 0.178  0.365 | N/A  N/A | N/A  N/A |  |
| ASDAS-LDA (<2.1)  *M6 and M12 vs.*  *persistent ASDAS ≥2.1*  *M6 or M12 vs.*  *persistent ASDAS ≥2.1* | N/A  N/A | N/A  N/A | 2.7 (1.4, 5.3)  1.2 (0.7, 2.2) | **0.004**  0.497 |  |

BASFI=Bath Ankylosing Spondylitis Functional Index; OR=Odds Ratio; CI=Confidence Interval; M=Month; BASDAI= Bath Ankylosing Spondylitis Disease Activity Index; ASDAS=Ankylosing Spondylitis Disease Activity Score; ID=Inactive Disease; LDA=Low Disease Activity. Bold P-values denote statistical significance.

Multivariable Analysis 1: primary analysis; Multivariable Analysis 2: secondary analysis including ASDAS-LDA instead of ASDAS-ID.
